# Palliative Care Needs Assessment in NY Nursing Homes: Psychiatric Symptoms

**DOI:** 10.1093/geroni/igaf122.1699

**Published:** 2025-12-31

**Authors:** Molly Nowels, Rose Carlson, Mark Unruh, M Carrington Reid, Catherine Riffin, Manali Saraiya, Evan Plys, Daniel Shalev

**Affiliations:** Weill Cornell Medicine, New York, New York, United States; Weill Cornell Medicine, New York, New York, United States; Joan and Sanford I. Weill College of Medicine, Cornell University, New York, New York, United States; Weill Cornell Medicine, New York, New York, United States; Weill Cornell Medicine, New York, New York, United States; New York-Presbyterian Hospital, New York, New York, United States; Massachusetts General Hospital, Boston, Massachusetts, United States; Weill Cornell Medicine, New York, New York, United States

## Abstract

Palliative care is specialized medical care aimed at improving quality of life for individuals with serious illnesses. Serious illness is prevalent among nursing home (NH) residents but integration of palliative care in NHs remains limited. Psychiatric comorbidities predict NH placement among older adults with serious illnesses, so the psychiatric domain of palliative care may be particularly critical in this setting. We conducted a palliative care needs assessment survey of 597 staff across seven NHs in New York. Our survey assessed staff experiences with specific domains of palliative care. Here, we present findings pertaining to the psychiatric components of palliative care: what proportion of respondents’ patients would benefit from psychiatric symptom management, the frequency with which respondents addressed psychiatric symptoms, and respondents’ comfort managing psychiatric symptoms. Over half of respondents (60.5%) identified psychiatric symptom management needs in at least half of their patients. Nearly half of respondents treated psychiatric symptoms at least weekly (49.2%) and felt comfortable treating psychiatric symptoms (48.7%). Staff with professional palliative care experience were likelier than those without to identify psychiatric needs in their patients (aOR 1.62 [1.07, 2.43]), treat psychiatric symptoms at least weekly (aOR 1.97 [1.29, 3.01]), and report comfort with treating psychiatric symptoms (aOR=1.54 [1.01, 2.35]). Professional experience in palliative care was was associated with increased recognition of psychiatric symptoms, and increased frequency and comfort in managing these symptoms. This suggests that workforce development in palliative care for NH staff may improve capacity in managing challenging psychiatric comorbidities.

